# Supplementary material for: Flexibility of Oxidized and Reduced States of the Chloroplast Regulatory Protein CP12 in Isolation and in Cell Extracts
Source: Biomolecules. 2021 May 8;11(5):701. doi: 10.3390/biom11050701 (PMC8151241; doi:10.3390/biom11050701)
Supplement: Supplementary file 1 [file biomolecules-11-00701-s001.zip › biomolecules-1186899-supplementary.pdf]

Supplementary materials

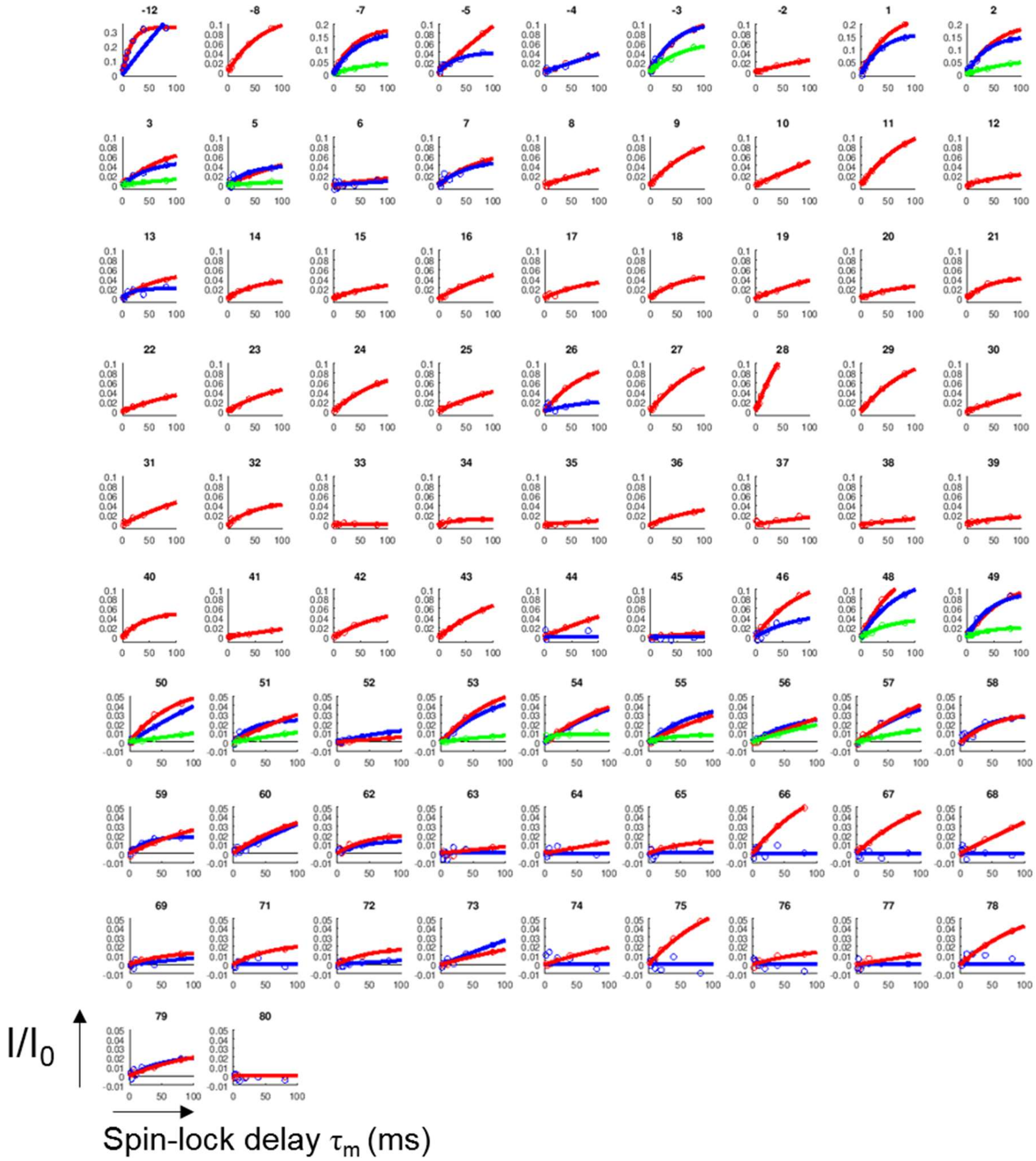

Figure S1. The signal intensity as a function of the spin-lock delay in the CLEANEX-PM experiments for $CP12_{red}$ at pH 7 (red), $CP12_{ox}$ at pH 7 (blue), $CP12_{ox}$ at pH 6 (green) for all CP12 residues. The fits are performed as indicated in the Material and Methods section. The number on each plot from 1 to 80 corresponds to the start (S) and to the end (D) of the amino acid sequence of the mature protein. For $CP12_{ox}$, all rates at pH 6 are significantly lower than those at pH 7, indicating that the exchange falls in the EX2 regime. This allows the determination of ΔG of folding.

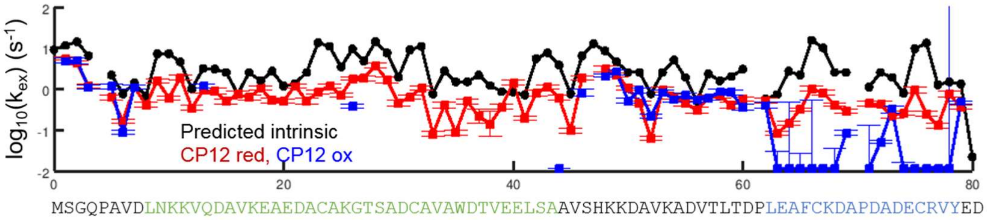

Figure S2. Solvent-amine intrinsic proton exchange rates calculated using Sphere (black) and solvent-amide proton exchange rates measured using CLEANEX-PM experiment on $CP12_{red}$ (red) and on $CP12_{ox}$ (blue).

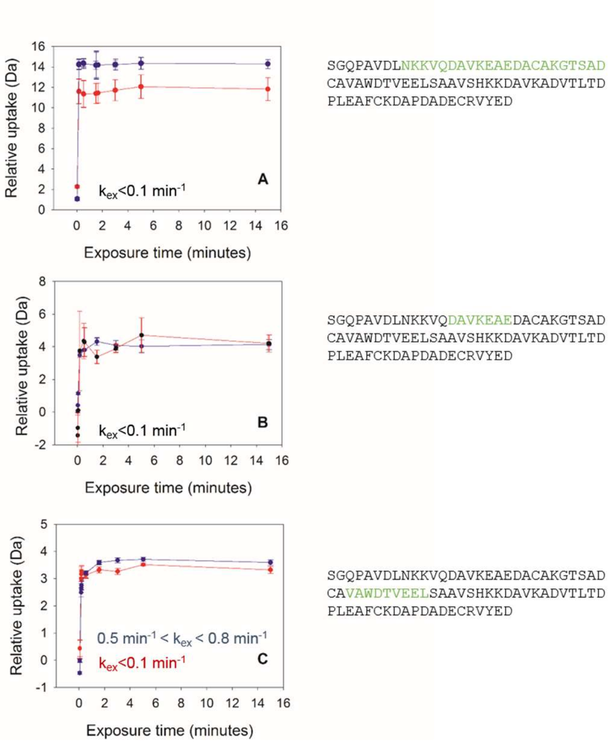

Figure S3. Relative uptake in mass of three peptides corresponding to the N-terminal region upon mixing with Deuterium of $CP12_{red}$ (red) and $CP12_{ox}$ (blue). The very rapid increase in mass confirms the dynamic nature of this region. The peptides are highlighted in green next to the corresponding graph.

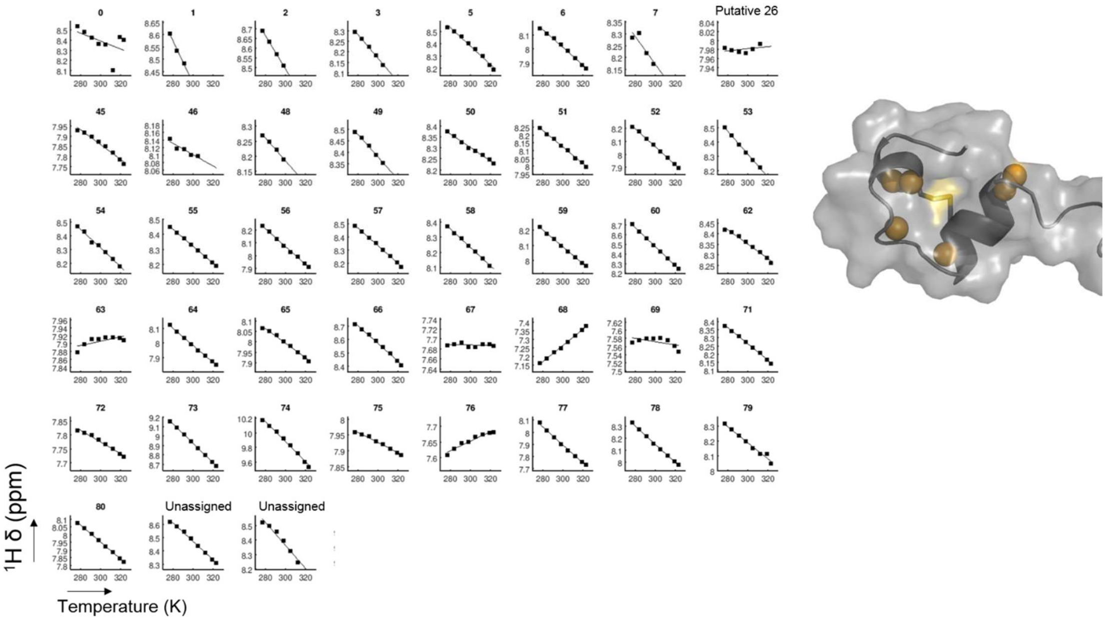

Figure S4. Temperature dependence of the proton chemical shift for the amine proton of all $CP12_{ox}$ residues. The fits are performed as indicated in the Material and Methods section. The structure on the right is that of the $CP12_{ox}$ C-terminal helical turn, with the amide protons that have a non-linear temperature dependence of their chemical shift highlighted in orange. This indicates that the N-terminal interconversion influences the C-terminal residues chemical shifts. The number on each plot corresponds to the amino acid position on the sequence of the protein.

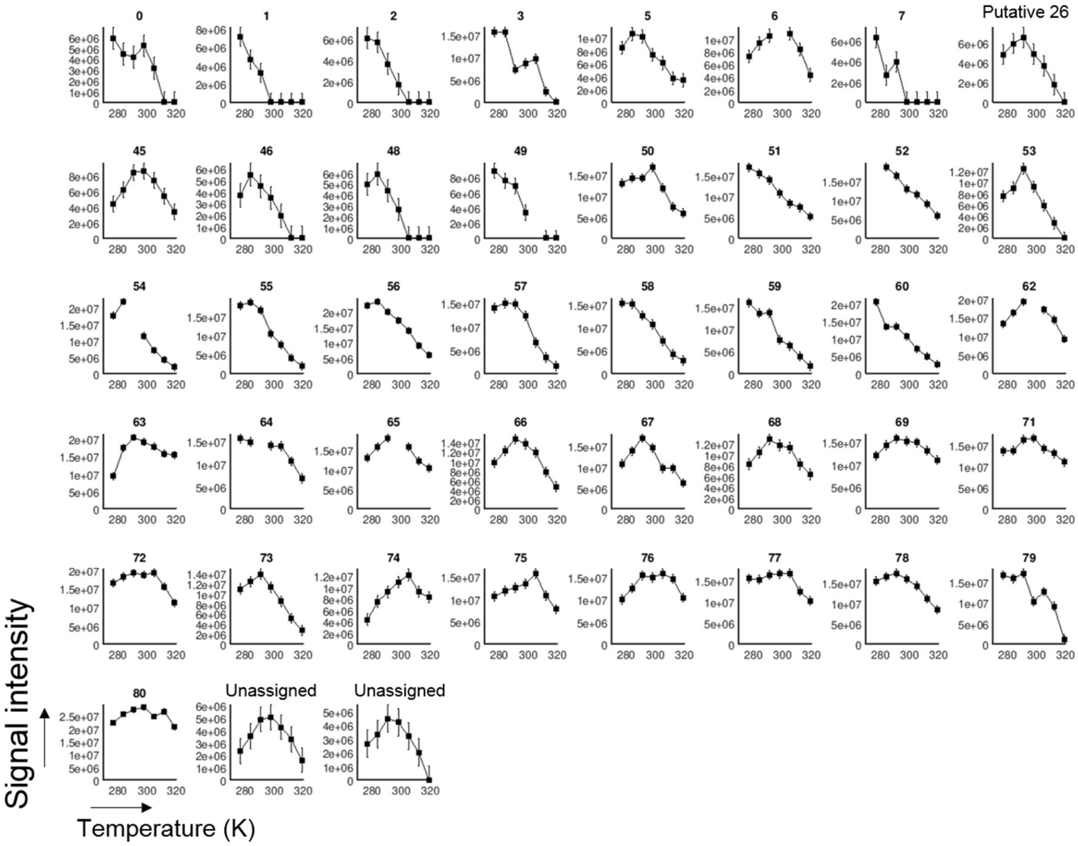

Figure S5. The temperature dependence of the ^{1}H-^{15}N signal intensity for the resonances of all CP12$_{ox}$ residues. The number on each plot corresponds to the amino acid position on the sequence of the protein.

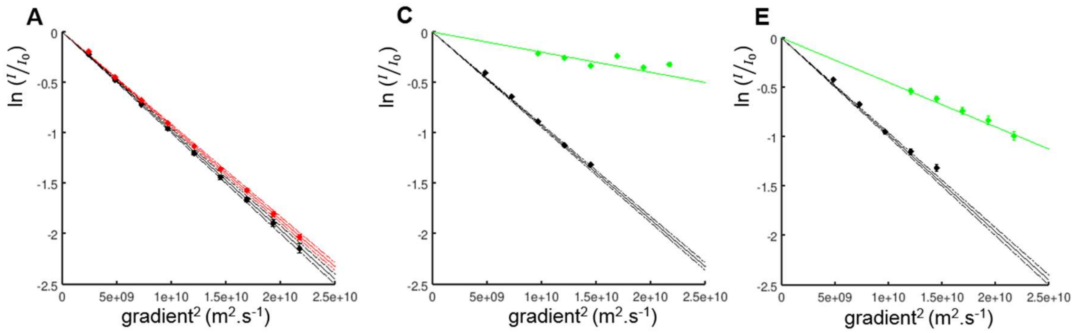

B	D (10^{-11} m^2.s^{-1})	R_h (nm)
Isolated $CP12_{ox}$	9.8 ± 0.2	1.9 ± 0.03
Isolated $CP12_{red}$	9.3 ± 0.15	2 ± 0.02

D	D (10^{-11} m^2.s^{-1})	R_h (nm)
Isolated $CP12_{red}$	9.3 ± 0.15	2 ± 0.02
$CP12_{red}$ + *C. reinhardtii* cell extract (light)	< 2	

F	D (10^{-11} m^2.s^{-1})	R_h (nm)
Isolated $CP12_{ox}$	9.8 ± 0.2	1.9 ± 0.03
$CP12_{ox}$ + *C. reinhardtii* cell extract (dark)	4.5 ± 0.5	

Figure S6. Hydrodynamics of $CP12_{ox}$ and $CP12_{red}$ measured by DOSY-NMR. A: STE on isolated $CP12_{ox}$ (black) and $CP12_{red}$ (red). **C:** XSTE on $CP12_{red}$ isolated (black) and in the presence of cell extract (green). **E:** XSTE on $CP12_{ox}$ isolated (black) and in the presence of cell extract (green).On the graphs A, C and E: the x-axis is the square of the gradient strength and on the y axis is the normalized intensity on a ln scale (refer to Material and Methods section). A linear dependency indicates a homogenous monomeric state of the protein. The tables of B, D and F show the measured diffusion coefficient from the data shown in A, C and E respectively, as well as the hydrodynamic radius for isolated CP12.

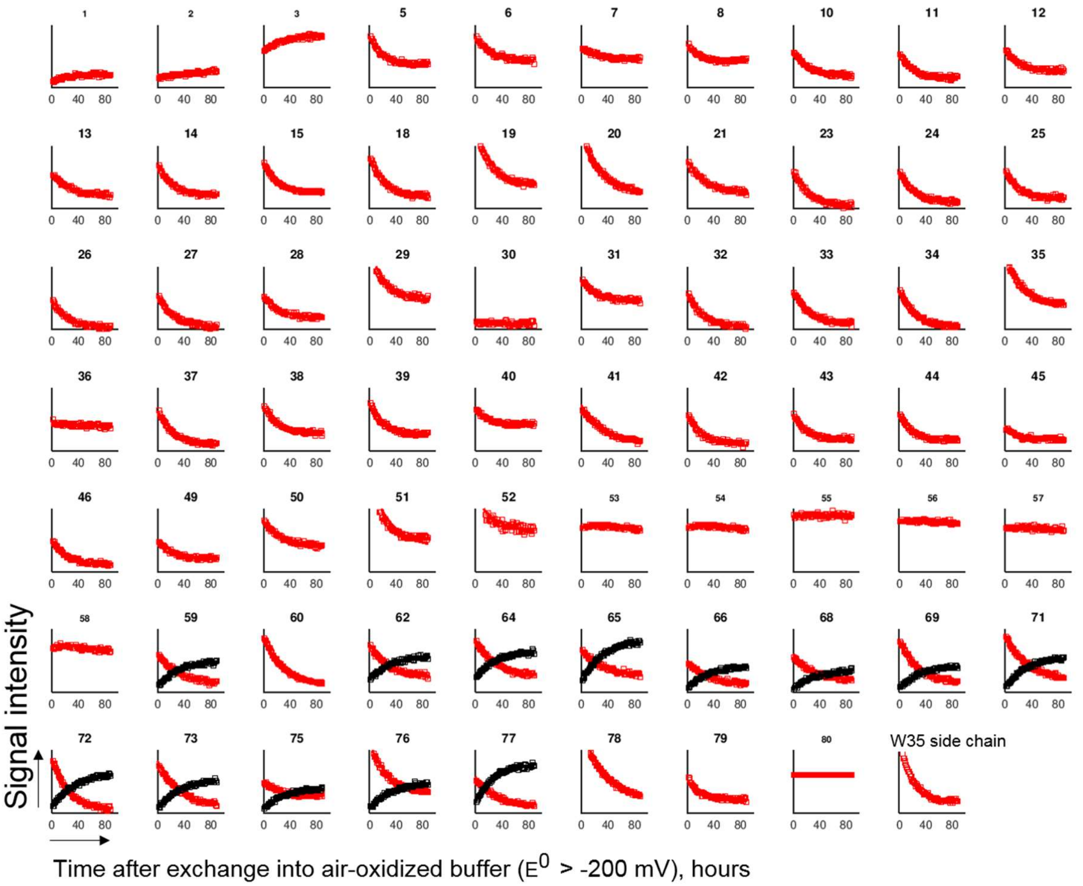

Figure S7. Real time monitoring of the oxidation for all CP12 residues. Red: Intensities of resonances of $CP12_{red}$. Black: Intensities of resonances of $CP12_{ox}$.

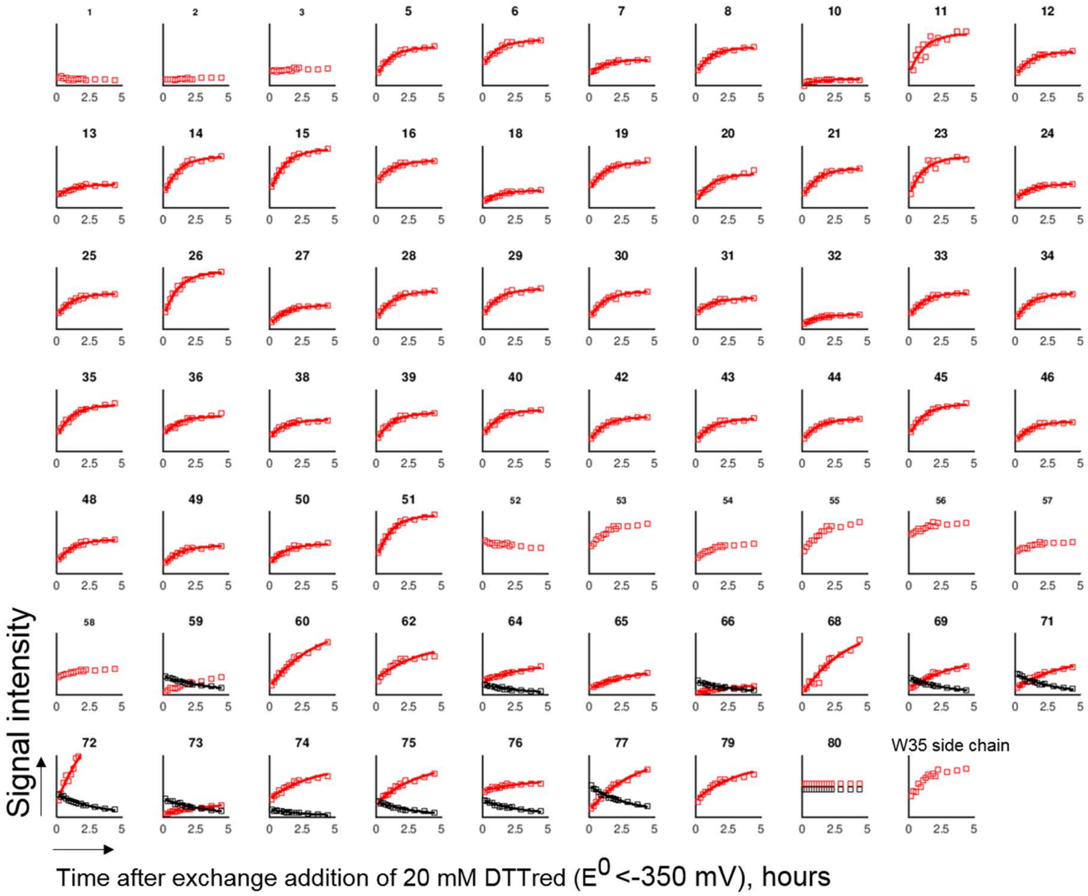

Figure S8. Real-time monitoring of the reduction for all CP12 residues. Red: Intensities of resonances of $CP12_{red}$. Black: Intensities of resonances of $CP12_{ox}$.
